# An Environmentally Benign Solvent for the Cationic Polymerization of Low Ceiling Temperature Polyaldehydes

**DOI:** 10.3390/polym17233210

**Published:** 2025-12-02

**Authors:** Jose C. Lopez Ninantay, Anthony C. Engler, Paul A. Kohl

**Affiliations:** 1 School of Chemical and Biomolecular Engineering, Georgia Institute of Technology, Atlanta, GA 30332, USA; jose.lopez@gatech.edu; 2 Cain Department of Chemical Engineering, Louisiana State University, Baton Rouge, LA 70803, USA; acengler@lsu.edu

**Keywords:** poly(phthalaldehyde), aldehyde copolymers, low ceiling temperature, green chemistry

## Abstract

The synthesis of phthalaldehyde-based polymers has exclusively been carried out in dichloromethane, which causes environmental problems due to its halogen content and ozone-depleting attributes. In this study, an alternative solvent for the polymerization of *o*-phthalaldehyde-based polyaldehydes is disclosed. Ethyl acetate, a solvent that is widely used in consumer products, dissolves a sufficient amount of reactants and polymer product at the reaction conditions, −86 °C, to provide a comparable yield to synthesis in dichloromethane. A significant learning from this study is that the reaction solvent does not have to fully dissolve all the reactants and products to produce stable polymer, compared to dichloromethane, which fully dissolves reactants and products. The polymer product precipitated from the ethyl acetate solution as the polymer formed. Although the reactants and products were not fully soluble in ethyl acetate, they retained sufficient mobility to allow the catalyst to initiate polymer chains and achieve molecular weights as high as 83.4 kg/mol. The synthesis of cyclic copolymers from *o*-phthalaldehyde and aliphatic aldehydes is also possible in ethyl acetate if the catalyst is added at a temperature below the ceiling temperature of the monomers and above the point where they crystallize from solution.

## 1. Introduction

Self-immolative polymers are presented as a possible means to close the plastic recycling loop [1,2,3]. Compared to traditional, hard-to-recycle plastics, significantly less energy is needed to trigger depolymerization of low ceiling temperature (Tc) polymers back to monomer, which can then be collected and reused in the synthesis of virgin polymer. Polyaldehydes based on *o*-phthalaldehyde (oPA) represent an example of a self-immolative polymer that can be reused in this way for various engineering applications [4,5,6,7,8,9].

The homopolymer of oPA, poly(phthalaldehyde) (PPA), has been prepared in dichloromethane (DCM) [10,11,12,13]. The polymerization mechanism at low temperature and the structure of the polymers have previously been discussed [10,11,12,13]. DCM is derived from oil, a non-renewable resource, and is an environmental hazard because it is an ozone-depleting halogen and suspected human carcinogen [14,15,16]. As a result, future industrial regulations of DCM handling and disposal may also complicate the manufacturing of PPA. This presents a conundrum for synthesizing phthalaldehyde-based self-immolative polymers for plastic recycling or other uses because a hazardous, non-renewable solvent is required in their synthesis. Finding a greener, alternative solvent for the polymerization of oPA is critically important.

In this study, the impact of the reaction solvent on the polymerization of oPA-based polymers is presented. More environmentally friendly solvents were investigated to improve the environmental footprint for fully recyclable polyaldehydes. Further, it has been found that the solvent does not need to fully dissolve the reactants and polymer product, which is challenging at the reaction temperature, ca. −86 °C, to produce stable, high molecular weight PPAs. Only limited solubility is needed to polymerize oPA polymers.

## 2. Materials and Methods

oPA (98%) was obtained from Tokyo Chemicals Incorporated (TCI) and distilled in vacuo (ca. 1.3 kPa) at 135 °C to produce vibrant yellow crystals. Boron trifluoride (BF_3_)-etherate (ca. 48% BF_3_) was obtained from Acros Organics (Geel, Belgium). Anhydrous DCM and anhydrous ethyl acetate (EtOAc) were obtained from EMD Millipore (Burlington, MA, USA). Cyclopentyl methyl ether (CPME, 99.5% extra dry), methyl acetate (MeOAc, 99+% extra dry), isobutyl acetate (iBuOAc, 98%), valeronitrile (99%), dimethyl isosorbide (DMI, 99%), butyl acetate (nBuOAc, 99% extra dry), and 2-methyltetrahydrofuran (mTHF, 99+% extra dry over molecular sieves) were obtained from Thermo Scientific (Waltham, MA, USA). Isoamyl acetate (iAmOAc) was obtained from TCI (Portland, OR, USA). Anhydrous toluene (Tol) was obtained from Alfa Aesar (Ward Hill, MA, USA). Anhydrous acetone, anhydrous methyl t-butyl ether (MTBE), and ethanal (EA, 99%) were obtained from Sigma-Aldrich (St. Louis, MO, USA) and used as-is. Propyl acetate (PrOAc, 99%) and pyridine (98%) were obtained from Beantown Chemical (Hudson, NH, USA). Diethyl ether (HPLC grade, not stabilized) and tetrahydrofuran (THF) were obtained from BDH Chemicals (Dawsonville, GA, USA). Gaseous CO_2_ (99.9%, bone dry) was obtained from Airgas (Atlanta, GA, USA).

Homopolymers of oPA were made by adding the monomer, solvent, and catalyst to the reaction vessel in a glovebox with a nitrogen atmosphere. The monomer concentration was 0.745 M, unless otherwise stated. The molar ratio of monomer-to-catalyst was 500-to-1 for all polymerizations. The monomer and solvent were added in the same nitrogen-filled glovebox for oPA copolymers. However, the catalyst was added to the mixture after cooling to avoid the oPA trimerization, which is known to occur with aliphatic aldehydes. The reaction vessel was then sealed and transferred to a constant temperature freezer at −86 °C. The polymerization was allowed to proceed for a set amount of time, after which the reaction was quenched by the injection of a 0.1 M pyridine in THF solution at a molar ratio of 50 moles of pyridine to 1 mole of catalyst. The quenched reaction was then precipitated dropwise into a volumetric excess of MeOH, unless otherwise stated. The resulting white powder was isolated by vacuum filtration. The product was dried in vacuo (ca. 1.3 kPa) for 24 h. Yield was calculated as the gravimetric ratio of isolated polymer product to monomer.

Polymer molecular weight and molar mass dispersity were obtained via gel permeation chromatography (GPC) on a Shimadzu GPC instrument with a Shodex KF-805 column (Shimadzu, Tokyo, Japan) with THF as the eluent (1 mL/min at 30 °C) using polystyrene standards. The elution time was calibrated with polystyrene standards. Copolymer composition was measured by ^1^H-nuclear magnetic resonance (NMR) spectroscopy on a Bruker Avance III (400 MHz) (Bruker, Billerica, MA, USA) by comparing integrals of acetal peaks from each monomer. The spectra were collected using deuterated chloroform as the solvent and its residual solvent peak (δ = 7.26 ppm) as the reference for chemical shifts.

## 3. Results and Discussion

Very few solvents have a freezing point below −86 °C (i.e., the reaction temperature) the polymerization temperature and fully dissolve the monomer and catalysts at this temperature, which is the traditional solvent objective. Table 1 is a list of the solvents surveyed for oPA polymerization at −86 °C using BF_3_-etherate catalyst. These solvents dissolve the oPA monomer at room temperature at the target concentration (0.745 M) and have melting points below −86 °C. Their solvatomagnetic basicity parameter (*β*) and respective PPA yield for a 3 h. reaction time are also reported in Table 1.

The solvents with a high solvatomagnetic basicity value (i.e., >0.52) had no product yield, which is consistent with the concept that if the solvent is too basic, it would compete for interactions with the Lewis acid catalyst and disrupt the polymerization mechanism and kinetics. Of the solvents with a low solvatomagnetic basicity value (i.e., <0.58), only four of the six (DCM, Tol, MeOAc, and EtOAc) had measurable PPA yield. Acetone and MeOAc showed visual signs of interaction with the catalyst because their color changed when BF_3_-etherate was added to the reaction mixture. This was further confirmed by ^19^F NMR (SI), where the fluorine peak shifted from the BF_3_-etherate value, showing an interaction between BF_3_ and the solvent.

The product yield in DCM was excellent (i.e., >90%), as expected from previous studies [11,12]. No discernable differences were found between the physical properties of the polymer synthesized in the different solvents. That is, the solvents appear to act only as a medium for the reaction and do not participate in the reaction. The only other solvent to give a high PPA yield was EtOAc. This was not expected because EtOAc is reportedly a poor solvent for PPA [19,20], and precipitation of the polymer product was observed. The reaction in EtOAc appears to be a pseudo-dispersion polymerization. That is, the reactants and products are not fully dissolved and dispersed in the reaction solvent. Interestingly, precipitation of the PPA product from the reaction mixture during the course of the reaction did not affect the product yield. The limiting factor for yield in EtOAc was likely the mobility of monomer species needed for chain propagation. It appears that the catalyst was able to interact with the monomer and the growing polymer strands even though they had limited solubility (i.e., formed a pseudo-dispersion).

Apart from providing the required mechanistic conditions to produce excellent yield, the pseudo-dispersion polymerization in EtOAc presents a unique opportunity to improve the catalyst removal from the reaction mixture after the polymerization is complete (i.e., quenching), which is critical to producing a long shelf-life polymer product [11,21]. The traditional quenching method uses pyridine to remove the catalyst, which relies on proper mixing within the viscous polymer solution. Conventionally, the resulting solution is precipitated into MeOH and vacuum filtered to obtain the solid polymer product. The solid is then redissolved in THF and repeatedly precipitated into MeOH or hexane until the desired purity is obtained. 

Polymer samples were synthesized from EtOAc solvent using alternative quenching methods, shown in Table 2. The resulting polymers were sealed in vials and aged in an oven at 45 °C. The number of days the polymer survived this accelerated aging test is an indication of the amount of residual catalyst in the polymer product. The number of days shown in Table 2 was determined by visual observation because once degradation occurs, monomer can be easily observed as a result of its low ceiling temperature.

The stability of the PPA product using the traditional quenching method, pyridine and THF, was consistent with previous results [11]. Additional washing of the polymer with methanol and pyridine did not improve the stability of the polymer. Washing with only methanol improved the stability by nearly 50% when compared to the traditional method. This is likely due to pyridine-BF_3_ being a relatively weak Lewis acid-base complex and thus not a perfect quenching method. The complex may not fully partition to the methanol phase, allowing it to contaminate the PPA product. 

Solid CsF was used as a solid quenching agent because it reacts with BF_3_ to form CsBF_4_, a filterable solid. The CsF-quenched polymer showed the lowest stability. This is not surprising because CsF is not soluble in EtOAc and any CsBF_4_ product formed would only be on the CsF surface. Although it did not provide the most effective BF_3_ removal, the CsF quenching method is interesting because enough BF_3_ was removed for the polymer to have measurable stability at room temperature. A solid-state BF_3_ removal method may be useful in cases where liquid additions are inconvenient.

**Figure 1:**
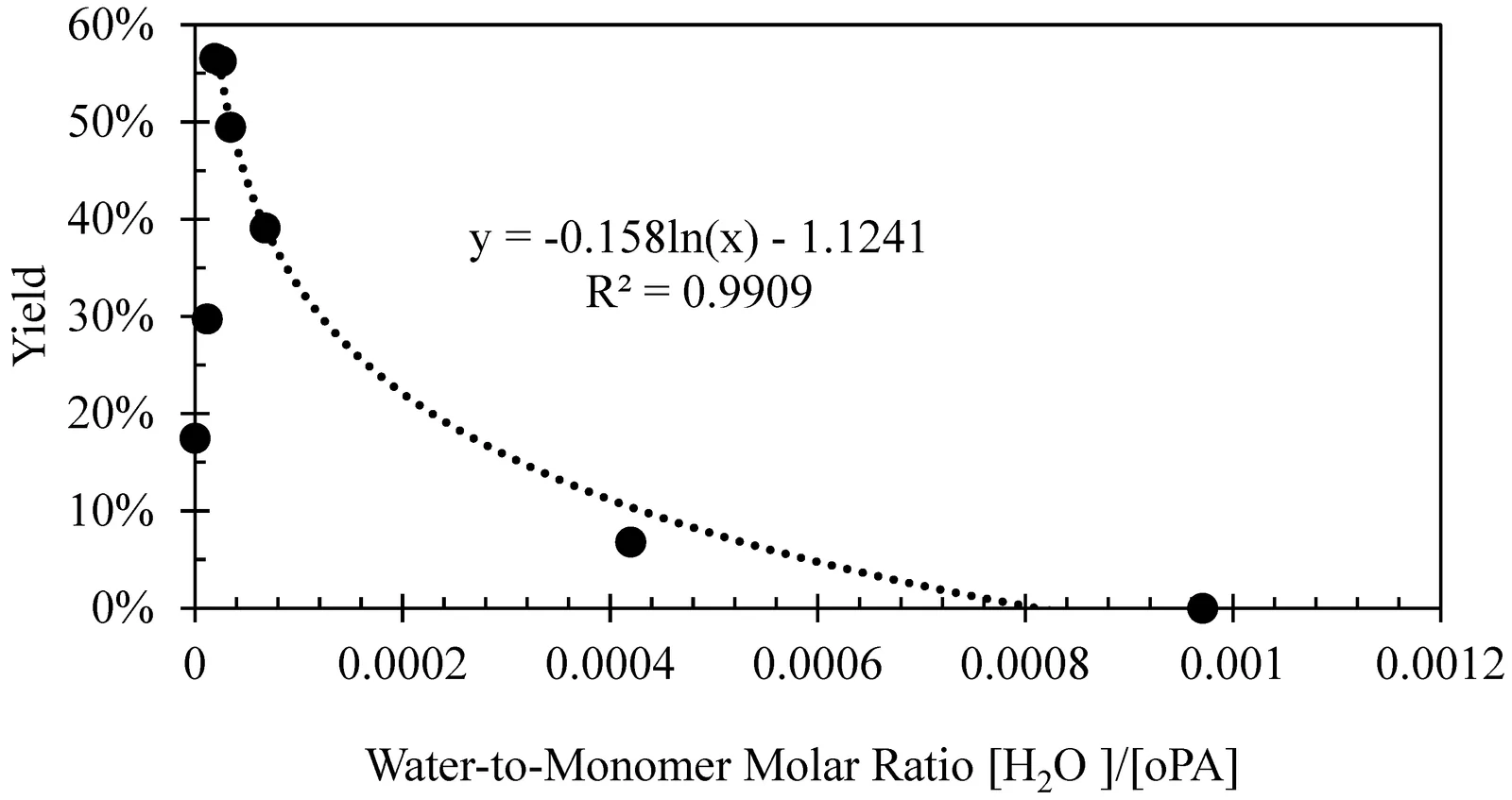
Yield of oPA polymerizations in EtOAc as a function of the ratio of moles of water added to the moles of oPA monomer.

The sensitivity of the oPA polymerization in EtOAc to residual water was investigated. The reactions are carried out in a nitrogen-purged glovebox with a very small residual water background concentration, which could affect the catalyst performance. Water was intentionally added to the polymerization mixture using EtOAc as the solvent. The yield as a function of the water added to the reaction mixture is plotted in Figure 1. The amount of water is reported with respect to the moles of monomer. The PPA yield decreased at higher water concentrations. In addition, it appears that a trace of water is beneficial for PPA product yield, as seen by the initial increase in yield in Figure 1. This co-catalyst type of behavior with water has been observed previously [22,23,24]. The logarithmic decline is likely explained by a competition between water and monomer, where the catalyst becomes inactive in the oPA polymerization reaction.

The efficacy of another catalyst for oPA polymerization in EtOAc vs. DCM solvent was also investigated. An ionic liquid containing a bridging chloride [Al_2_Cl_7_^−^] anion was selected because of its ease of use and relative activity in the polymerization of oPA [11]. Table 3 compares the yield and molecular weight obtained for BF_3_ and [Al_2_Cl_7_^−^] catalysts in EtOAc and DCM in back-to-back experiments, keeping all other polymerization conditions constant (including water content, monomer purity, time, and temperature). The products were quenched with pyridine/THF.

The yields for BF_3_ in DCM and EtOAc were comparable, about 90%. However, the yields with [Al_2_Cl_7_^−^] were measurably different. The yield in EtOAc was better than in DCM, although the product molecular weight was about half of that in DCM. This is reasonable because the PPA polymer precipitated out of EtOAc during synthesis so that its interaction time with the catalyst in solution was shorter. Previously, it was shown that the molecular weight of the PPA polymer grows in solution by ring fusion [22].

EtOAc is an effective new solvent for oPA polymerization because the catalyst could be added to the reaction solution before the mixture was cooled to the reaction temperature. This allowed the polymer to begin formation as the temperature was lowered to −86 °C which is desirable when the solvent does not fully dissolve the reactants or products at the reaction temperature. Copolymers of oPA with aliphatic aldehydes cannot be synthesized in this way (i.e., adding the catalyst before cooling) because the formation of an aliphatic aldehyde trimer side-product (trioxane) inhibits copolymer formation [25,26,27]. Instead, the catalyst must be added to the reaction solution after cooling below the ceiling temperature of the copolymer [26,28,29]. During cooling, there is a balance between minimizing trimer formation and preventing monomer precipitation. 

Polymerization of oPA and ethanal (EA) was performed to investigate the viability of oPA/aliphatic aldehyde synthesis in EtOAc. EtOAc and DCM solutions with oPA and EA monomers were prepared and sealed in a glovebox. The initial mixture was 80 mol% EA and 20 mol% oPA with a total monomer concentration of 0.85 M. An additional solution was prepared with no solvent (i.e., bulk polymerization), relying on the solubility of oPA in EA monomer (80 mol% EA) that remained liquid down to −123 °C. The vessel was placed in a −86 °C freezer and monitored for the point when the monomer precipitated from the EtOAc solution. BF_3_-etherate was added at this point, and the reaction was allowed to proceed for 96 h. The extended time was used to ensure the reaction had sufficient time for maximum consumption of monomer. Table 4 shows the results of these polymerizations.

The polymerizations shown in Table 4 yielded polymer in each experiment. The polymer obtained from EtOAc solvent was lower molecular weight than that from DCM solvent, which is consistent with the PPA homopolymerization; however, the overall copolymer yield was comparable. The reaction in EA yielded some polymer, although it had lower yield and molecular weight than those with solvent. This suggests that the polymerization solvent needs to dissolve a portion of the reactants or products to effectively produce copolymer, although minimal dissolution can still produce measurable yield. The EA incorporation for the three polymerizations appears to be determined by the initial EA concentration, and thus the reactions gave similar EA incorporation for the resulting polymers.

**Figure 2:**
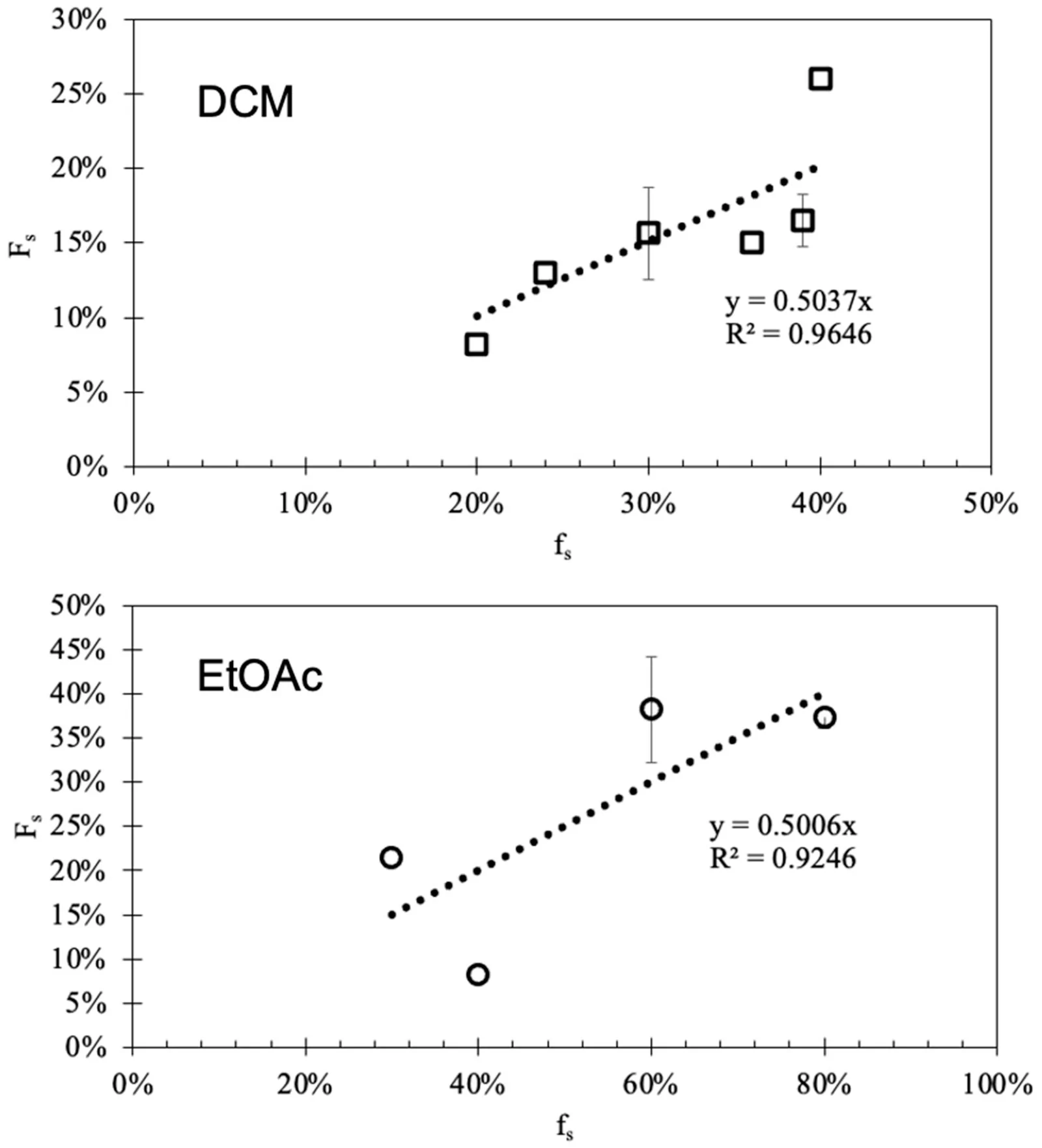
Mole percent EA in the product, Fs (*y*-axis), vs. mole fraction of EA in the EA/oPA reaction mixture, fs (*x*-axis), for reactions using DCM solvent (**top**) and EtOAc solvent (**bottom**). The dashed line is the least square regression fit to the data (squares in the top figure, circles in the bottom figure).

The EA incorporation into the EA/oPA copolymer was further investigated in DCM and EtOAc reaction solvents. The mole fraction of EA in the polymer product (Fs) was studied as a function of the mole fraction of EA in the EA/oPA reaction mixture (fs) [26]. Figure 2 shows the results for a 1 h polymerization with a total monomer concentration of 0.4 M. It is shown that the mole percent EA in the product increased with the mole percent EA in the feed for both solvents. Further, it has also been shown that higher EA content can be achieved in EtOAc than DCM.

Synthesis in a solvent that is gaseous at ambient conditions is desirable for bulk manufacturing because the solvent could be easily removed by evaporation. Carbon dioxide (CO_2_) has been reported as a green solvent for polymer processes [30,31]. The impact of CO_2_ as the solvent on the polymerization of oPA was investigated in a high-pressure Parr reactor. The results of a series of reactions are shown in Table 5 along with a DCM control.

The lack of stirring in the Parr reactor may explain the slight reduction in yield for DCM when compared to a round-bottom flask where stirring was easily performed. When a CO_2_ overpressure of 100 kPa was added to a reaction in DCM, the yield decreased further. The CO_2_ dissolved in the DCM slightly interrupted the polymerization, so an additional polymerization was performed in liquid CO_2_ (−56.5 °C and 518.5 kPa). The temperature was maintained at −56.5 °C to keep CO_2_ liquid. The polymer yield for the liquid CO_2_ reaction was small. The presence of polymer was confirmed by ^1^H NMR. A solid sample of this reaction was taken prior to quenching with THF and pyridine and analyzed using ^1^H NMR. The most prominent peak observed apart from oPA monomer, DCM, and BF_3_-etherate, was at 5.32 ppm. This peak can be explained by the formation of phthalide [32,33], which renders oPA inactive for any polymerization. The concentration of phthalide could have been enough to react with the BF_3_ catalyst and stop the polymerization.

## 4. Conclusions

A green alternative to the use of DCM solvent for the cationic polymerization of oPA was found: EtOAc. Polymer precipitated out of solution as it formed in EtOAc, which makes EtOAc a potentially reusable solvent for continuous synthesis of PPA. The partition between reactant and product also allowed for more effective removal of the catalyst after polymerization, increasing the shelf-life stability of the material compared to PPA synthesized in DCM. Copolymers with aliphatic aldehydes (ethanal) are also shown to be possible in EtOAc by adding the catalyst at the optimum temperature to balance the monomer solubility and the competing aliphatic aldehyde trimerization reaction.

## Figures and Tables

**Table 1:** Solvents used in this study for oPA polymerization with BF_3_ catalyst at −86 °C (see Section 2 Methods section for detailed synthetic procedures), their solvatomagnetic basicity parameter β [17,18], and their respective maximum yield of PPA product.

Solvent	Solvatomagnetic *β*	Maximum Yield
DCM	0.0	98%
Tol	0.11	45%
Valeronitrile	0.46	None
Acetone	0.49	None
MeOAc	0.51	38%
EtOAc	0.52	98%
THF	0.58	None
nBuOAc	0.58	None
Diethyl ether	0.59	None
PrOAc	0.6	None
CPME	0.63	None
mTHF	0.66	None
MTBE	0.67	None
iBuOAc	-	None
DMI	-	None
iAmOAc	-	None

**Table 2:** Results of accelerated aging stability test for PPA polymers prepared using EtOAc and alternative catalyst quenching methods.

Quench Method	Stability (45 °C Oven)
MeOH Precipitation	60 days
MeOH + Pyridine Wash	41 days
MeOH Wash	86 days
CsF	28 days

**Table 3:** Yield, number average molecular weight, and molar mass dispersity for BF_3_ and [Al_2_Cl_7_^−^] in DCM and EtOAc.

	DCM			EtOAc		
Catalyst	Yield	M_n_ (kg/mol)	Ð	Yield	M_n_ (kg/mol)	Ð
BF_3_	87%	148	1.55	92%	74.6	1.67
[Al_2_Cl_7_^−^]	63%	61.4	1.58	96%	30.0	1.72

**Table 4:** Results from EA/oPA copolymerization reactions in three different solvents.

Solvent System	Ethanal Content	M_n_ (kg/mol)	Yield
DCM	26 mol%	64.0	38%
EtOAc	26 mol%	10.4	41%
Ethanal	25 mol%	3.8	5.2%

**Table 5:** Sample polymerizations conducted to study liquid CO_2_ as a reaction solvent.

Solvent	Yield	Mn (kDa)	Ɖ
DCM (Parr)	75.1%	91.7	1.35
DCM + CO_2_	67.5%	51.0	1.39
Liquid CO_2_	*Slight*	-	-

## Data Availability

The original contributions presented in this study are included in the article. Further inquiries can be directed to the corresponding author.
