# Correction: Lopez Ninantay et al. An Environmentally Benign Solvent for the Cationic Polymerization of Low Ceiling Temperature Polyaldehydes. *Polymers* 2025, *17*, 3210

**DOI:** 10.3390/polym18151835

**Published:** 2026-07-27

**Authors:** Jose C. Lopez Ninantay, Anthony C. Engler, Paul A. Kohl

**Affiliations:** 1 School of Chemical and Biomolecular Engineering, Georgia Institute of Technology, Atlanta, GA 30332, USA; jose.lopez@gatech.edu; 2 Cain Department of Chemical Engineering, Louisiana State University, Baton Rouge, LA 70803, USA; acengler@lsu.edu

One of the authors, Jared M. Schwartz, was removed in the original publication [1]. The corrected Author Contributions statement appears here.

Conceptualization, A.C.E. and P.A.K.; Validation, J.C.L.N. and A.C.E.; Formal analysis, J.C.L.N. and P.A.K.; Investigation, J.C.L.N., A.C.E. and P.A.K.; Resources, P.A.K.; Data curation, J.C.L.N. and P.A.K.; Writing—original draft, J.C.L.N.; Writing—review and editing, J.C.L.N., A.C.E. and P.A.K.; Supervision, A.C.E. and P.A.K.; Project administration, P.A.K. All authors have read and agreed to the published version of the manuscript.

The authors state that the scientific conclusions are unaffected. This correction was approved by the Academic Editor. The original publication has also been updated.
